# HuR Reduces Radiation-Induced DNA Damage by Enhancing Expression of ARID1A

**DOI:** 10.3390/cancers11122014

**Published:** 2019-12-13

**Authors:** Daniel Andrade, Meghna Mehta, James Griffith, Sangphil Oh, Joshua Corbin, Anish Babu, Supriyo De, Allshine Chen, Yan D. Zhao, Sanam Husain, Sudeshna Roy, Liang Xu, Jeffrey Aube, Ralf Janknecht, Myriam Gorospe, Terence Herman, Rajagopal Ramesh, Anupama Munshi

**Affiliations:** 1 Department of Radiation Oncology, The University of Oklahoma Health Sciences Center, Oklahoma City, OK 73104, USA; daniel-andrade@ouhsc.edu (D.A.); meghna-mehta@ouhsc.edu (M.M.); james-griffith@ouhsc.edu (J.G.); terence-herman@ouhsc.edu (T.H.); 2 Stephenson Cancer Center, The University of Oklahoma Health Sciences Center, Oklahoma City, OK 73104, USA; sangphil-oh@ouhsc.edu (S.O.); anish-babu@ouhsc.edu (A.B.); daniel-zhao@ouhsc.edu (Y.D.Z.); ralf-janknecht@ouhsc.edu (R.J.); rajagopal-ramesh@ouhsc.edu (R.R.); 3 Department of Cell Biology, The University of Oklahoma Health Sciences Center, Oklahoma City, OK 73104, USA; 4 Department of Pathology, The University of Oklahoma Health Sciences Center, Oklahoma City, OK 73104, USA; joshua-corbin@ouhsc.edu (J.C.);; 5 National Institute on Aging, National Institutes of Health, Baltimore, MD 21224, USA; desu@grc.nia.nih.gov (S.D.); myriam-gorospe@nih.gov (M.G.); 6 Department of Biostatistics and Epidemiology, The University of Oklahoma Health Sciences Center, Oklahoma City, OK 73104, USA; allshine-chen@ouhsc.edu; 7 Division of Chemical Biology and Medicinal Chemistry, University of North Carolina at Chapel Hill, Chapel Hill, NC 27599, USAjaube@email.unc.edu (J.A.); 8 Department of Molecular Biosciences, University of Kansas Medical Center, Kansas City, KS 66160, USA; xul@ku.edu; 9 Graduate Program in Biomedical Sciences, The University of Oklahoma Health Sciences Center, Oklahoma City, OK 73104, USA

**Keywords:** ARID1A, BAF250a, SWI/SNF, HuR, ELAVL1, post-transcriptional regulation, RNA-binding protein, TNBC, radiation

## Abstract

Tumor suppressor ARID1A, a subunit of the chromatin remodeling complex SWI/SNF, regulates cell cycle progression, interacts with the tumor suppressor TP53, and prevents genomic instability. In addition, ARID1A has been shown to foster resistance to cancer therapy. By promoting non-homologous end joining (NHEJ), ARID1A enhances DNA repair. Consequently, ARID1A has been proposed as a promising therapeutic target to sensitize cancer cells to chemotherapy and radiation. Here, we report that ARID1A is regulated by human antigen R (HuR), an RNA-binding protein that is highly expressed in a wide range of cancers and enables resistance to chemotherapy and radiation. Our results indicate that HuR binds *ARID1A* mRNA, thereby increasing its stability in breast cancer cells. We further find that ARID1A expression suppresses the accumulation of DNA double-strand breaks (DSBs) caused by radiation and can rescue the loss of radioresistance triggered by HuR inhibition, suggesting that ARID1A plays an important role in HuR-driven resistance to radiation. Taken together, our work shows that HuR and ARID1A form an important regulatory axis in radiation resistance that can be targeted to improve radiotherapy in breast cancer patients.

## 1. Introduction

The human antigen R (HuR), also known as ELAVL1 (embryonic lethal abnormal vision-like 1), is an RNA-binding protein that binds preferentially to AU- or U-rich elements (AREs) usually located in the 3′ untranslated region (UTR) of its target transcripts [1]. HuR is mainly localized in the nucleus, but after exposure to specific stresses translocates to the cytoplasm where it can influence the stability and/or translation of subsets of mRNAs [2]. Through these actions, HuR regulates several cellular processes, including cell proliferation, the response to DNA damage, and cell survival [3,4,5,6]. Elevated expression of HuR is found in tissues of virtually all forms of cancer, regulating the stability of many mRNAs that encode cancer-related proteins, thereby promoting survival, proliferation, angiogenesis, and invasion [7,8,9,10,11,12,13]. Several studies have reported that HuR contributes to resistance to chemotherapy in many types of cancers, including breast cancer [14,15,16,17]. We have recently shown that HuR facilitates radiation resistance in triple negative breast cancer (TNBC) by protecting from radiation-induced DNA damage [18]. Given the importance of HuR in cancer biology, it is necessary to get a better understanding of the broad regulatory activity of HuR in order to identify targets and mechanisms by which HuR orchestrates resistance to cancer therapies.

The SWI/SNF (switch/sucrose non-fermenting) complexes are ATPase-powered chromatin remodelers. They bind to specific positions on the DNA and mobilize nucleosomes by the energy generated through the ATPase activity of either BRM or BRG1 [19]. By altering the accessibility of transcription factors to promoters/enhancers, the SWI/SNF complex can modulate transcription positively or negatively [20,21]. Through this mechanism, the SWI/SNF complex plays essential roles in a variety of cellular processes such as differentiation, proliferation, and tumor suppression. SWI/SNF also plays critical roles in the DNA damage response by propagating DNA damage signals and facilitating access of DNA repair proteins to the DNA damage sites [22,23,24,25]. In yeast, SWI/SNF plays an active role in the repair of DNA double-strand breaks (DSBs), mainly by promoting homologous recombination [25,26,27]. Likewise, elements of the SWI/SNF complex have been shown to be required for non-homologous end joining (NHEJ) activity of DSBs in human cancer cells, suggesting that the SWI/SNF complex could favor resistance to therapeutic agents that induce DNA damage in tumor cells [28]. In fact, due to its role in DNA damage, some studies have highlighted the importance of targeting the SWI/SNF catalytic subunits to sensitize cancer cells to therapeutic agents [29,30].

The AT-rich interactive domain 1A (SWI-like) gene (*ARID1A*), which encodes the protein BAF250, a key component of the SWI/SNF complex, is usually described as a tumor suppressor. It is one of the most frequently mutated genes in many types of cancer, especially in ovarian, gastric, lung, and breast cancers [31,32]. As part of the SWI/SNF complex, ARID1A controls gene expression by directing the complex to specific target promoters [33,34,35,36]. ARID1A actively participates in the regulation of several cellular events. It controls cell cycle arrest by inducing expression of the cyclin-dependent kinase inhibitor 1A (p21^CDKN1A^) and by reducing the expression of E2F-responsive genes such as *CDC2* [37]. ARID1A also participates in DNA repair, a molecular function important for resistance to radiation and chemotherapy. It has been shown that ARID1A promotes NHEJ activity by facilitating the accumulation of Ku70/Ku80 proteins at DSBs, conferring resistance to UV, ionizing radiation, and cisplatin in lung and bone osteosarcoma cells [28]. ARID1A loss correlates with mismatch repair deficiency in endometrial cancer [38]. Loss of ARID1A leads to impaired checkpoint activation and repair of DNA DSBs, which sensitizes cells to DSB-inducing treatments, such as radiation and poly (ADP-ribose) polymerase (PARP) inhibitors. Thus, even though ARID1A is mainly described as a tumor suppressor, recent investigations point to the importance of targeting ARID1A in order to sensitize cancer cells to chemotherapy and radiation [28,34,39]. 

In this work, we uncover a direct interaction between the oncoprotein HuR and *ARID1A* mRNA and demonstrate that HuR post-transcriptionally promotes ARID1A expression. We also present evidence that genetic inhibition of ARID1A leads to accumulation of radiation-induced DSBs and sensitizes TNBC cells to radiation. In addition, forced expression of ARID1A re-establishes radioresistance in cells where HuR is genetically inhibited, suggesting that ARID1A plays an important role in HuR-driven resistance to radiation. These findings expand our understanding of the mechanisms by which HuR promotes radiation resistance, and underscore the value of targeting the HuR–ARID1A axis in order to enhance the effectiveness of radiotherapy for patients with breast cancer. 

## 2. Results

### 2.1. ARID1A mRNA Is a Novel HuR Target

**Figure 1:**
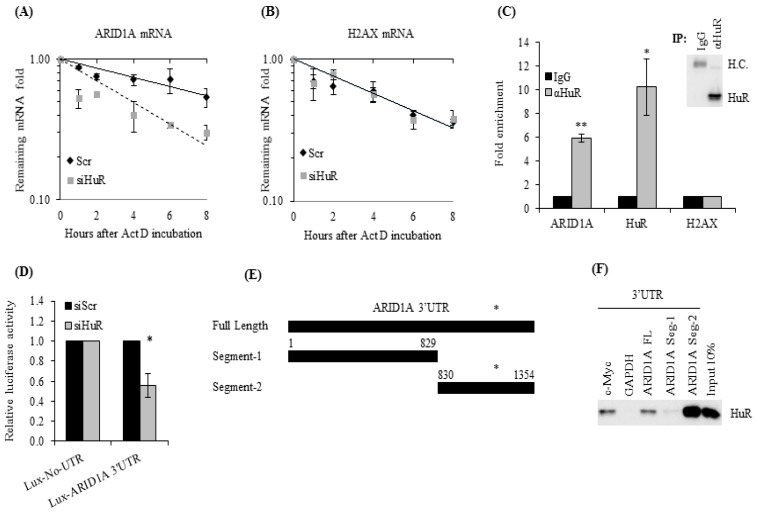
*ARID1A* is a bona fide human antigen R (HuR) target. (**A**) Twenty-four hours after transfection with siHuR, MDA-MB-231 cells were treated with Actinomycin D and collected at the indicated time points to evaluate their ARID1A mRNA levels by RT-qPCR. (**B**) *H2AX* mRNA levels were also evaluated as a negative control. (**C**) MDA-MB-231 cell lysates were used to immunoprecipitate HuR using an antibody against HuR or IgG. HuR immunocomplexes were subjected to RNA extraction, and the levels of *ARID1A* mRNA were measured by qRT-PCR. The levels of *HuR* and *H2AX* mRNAs were also measured as positive and negative controls, respectively. Inset shows HuR immunoprecipitation by HuR antibody, but not by IgG control. (**D**) Eight hours after transfection with HuR siRNA, MDA-MB-231 cells were transfected with a vector encoding either luciferase alone (Lux-No-UTR) or luciferase fused to the 3′UTR of *ARID1A* (Lux-ARID1A 3′UTR); 16 h later, luciferase activity was evaluated. (**E**) Three segments of *ARID1A* 3′UTR were cloned into a T7 containing vector to generate biotinylated RNA probes. Asterisk indicates regions of high probability for HuR binding. (**F**) These probes were incubated with MDA-MB-231 lysates, recovered with neutravidin-coated beads, and subjected to immunoblot analysis to evaluate the levels of HuR bound to the probes. Data represent the average of three independent experiments. Error bars represent SEM (standard error of the mean), * *p* ≤ 0.01 and ** *p* ≤ 0.001.

We previously showed that HuR expression promotes resistance to gamma radiation in TNBC cells, mainly by controlling the DNA damage response (DDR) [18]. In order to identify HuR targets in the DDR pathway, we performed microarray analysis of RNA molecules bound to HuR, obtained through ribonucleoprotein immunoprecipitation (RIP) assays. In the RIP assay, transcripts bound to HuR were isolated from lysates of irradiated and non-irradiated MDA-MB-231 cells, using an antibody against HuR (data not shown). Along with several other transcripts, the screening identified *ARID1A* mRNA as a potential novel HuR target in the DDR pathway during radiation. To test this observation, we first measured the half-life of *ARID1A* mRNA in the presence of normal and reduced HuR levels. Following treatment with Actinomycin D, which inhibits RNA polymerase II and thereby allows the measurement of half-lives of transcribed RNAs, total RNA was harvested at several time points and the levels of *ARID1A* mRNA were quantified by RT-qPCR analysis. In the presence of HuR, *ARID1A* mRNA had a half-life of >8 h (Figure 1A). In contrast, in HuR-silenced cells, *ARID1A* mRNA half-life was dramatically reduced to 3.9 h (Figure 1A). As a control, we measured the levels of *H2AX* mRNA, which is not a target of HuR, and found it to be equally stable in the presence or absence of HuR (Figure 1B). These results suggest that HuR regulates ARID1A expression by stabilizing *ARID1A* mRNA. To verify whether HuR truly associates with endogenous *ARID1A* mRNA, we performed an RIP analysis. For this, HuR was immunoprecipitated using an antibody against HuR and IgG as control (Figure 1C). We then measured the enrichment of *ARID1A* mRNA in the HuR RIP compared with the IgG RIP by RT-qPCR analysis. Enrichments in *HuR* and *H2AX* mRNAs were included as positive and negative controls, respectively. Compared with IgG control, *ARID1A* mRNA was enriched 6-fold in the HuR RIP sample, supporting the notion that HuR directly interacts with *ARID1A* mRNA (Figure 1C).

To further explore the interaction between HuR and *ARID1A* mRNA, we searched the public online database starBase v2.0, which systematically identifies RNA–RNA and protein–RNA interaction networks from 108 CLIP-Seq (PAR-CLIP, HITS-CLIP, iCLIP, CLASH) datasets generated by 37 independent studies [40,41]. starBase analysis not only confirmed that HuR binds *ARID1A* mRNA but also showed that it binds at multiple sites on the 3′UTR and the coding region of the *ARID1A* transcript (Supplementary Figure S1). We also searched another web server, RBPmap, for mapping binding sites of RNA-binding proteins. Consistently, this database also predicted multiple HuR binding sites at the *ARID1A* 3′UTR, further supporting our findings that HuR interacts with *ARID1A* mRNA ([42]; Supplementary Figure S1). 

The public online database starBase v2.0 and the web server RBPmap predicted multiple HuR binding sites at different places in *ARID1A* mRNA (Supplementary Figure S1). Although very different from each other, their predicted sites overlap at various sites on the 3′UTR of *ARID1A* mRNA, suggesting that this region could play an important role in the regulation of ARID1A expression by HuR. To test whether the *ARID1A* 3′UTR influenced the stability of *ARID1A* mRNA conferred by HuR, we used a reporter construct that expressed a chimeric RNA containing the luciferase coding region fused to the *ARID1A* 3′UTR. We then evaluated the expression of this construct in transfected cells by measuring luciferase activity. Cells expressing reduced HuR levels showed decreased levels of luciferase activity (~55%) compared with control cells, suggesting that HuR confers stability via the *ARID1A* 3′UTR (Figure 1D). Luciferase activity in cells transfected with a control vector that expressed luciferase without the *ARID1A* 3′UTR was not altered when HuR was silenced (Figure 1D). These results further support our findings that HuR regulates the stability of *ARID1A* mRNA by interacting with the *ARID1A* 3′UTR. 

To obtain further biochemical evidence that HuR binds the *ARID1A* 3′UTR, we synthesized biotinylated RNA probes corresponding to the full-length form of *ARID1A* 3′UTR (nucleotides 1–1354) and to the regions of low (1–829) and high (830–1354) probability of binding to HuR. High- and low-probability regions were arbitrarily defined by prediction overlap between the two databases and evolutionary conservation among 100 species (Figure 1E, Supplementary Figure S1). We also synthesized probes for the 3′UTR of *c-Myc* and *GAPDH* as positive and negative controls, respectively. The biotinylated probes were incubated with MDA-MB-231 whole-cell lysates and recovered with neutravidin-coated beads for Western blot analysis to measure HuR bound to the probes (Figure 1E,F). Similar to the positive control *c-Myc* RNA, HuR was found to bind to the full-length *ARID1A* 3′UTR. We also found that HuR bound poorly to the segment of low binding probability (1–829) and strongly to the segment of high binding probability (830–1354) of *ARID1A* 3′UTR. 

### 2.2. HuR Promotes ARID1A Expression

**Figure 2:**
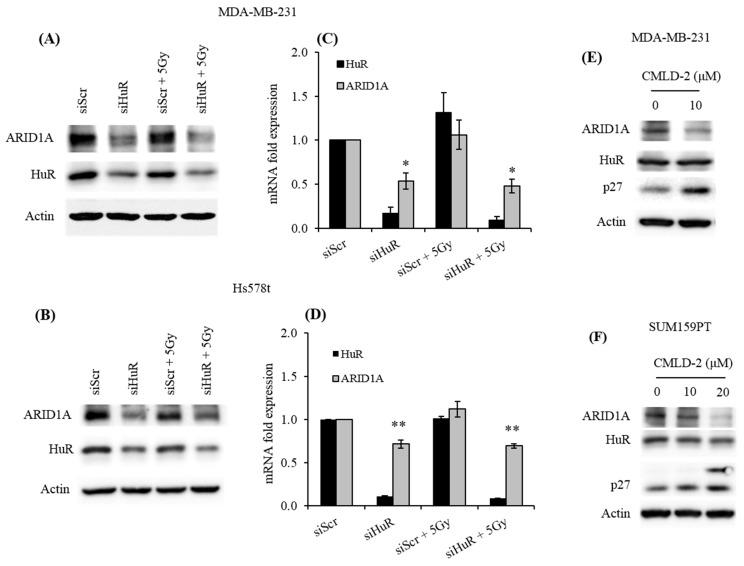
Genetic or pharmacological inhibition of HuR reduces ARID1A levels. Twenty-four hours after transfection with HuR siRNA, MDA-MB-231 and Hs578t cells were treated with a radiation dose of 5 Gy; 2 h later, they were evaluated for ARID1A expression by (**A**,**B**) Western blot or (**C**,**D**) (q) RT-PCR analyses. MDA-MB-231 and SUM159PT cells treated with HuR inhibitor, CMLD-2, for 24 h were collected to evaluate ARID1A expression levels. (**E**,**F**) As a control for HuR inhibition, p27 expression was also evaluated in MDA-MB-231 and SUM159PT following CMLD-2 treatment. Data presented are the average of three independent experiments. Error bars represent SEM (standard error of the mean), * *p* ≤ 0.01 and ** *p* ≤ 0.001.

We next evaluated the effect of modulating HuR expression on ARID1A levels in two breast cancer cell lines—MDA-MB-231 and Hs578t. First, we silenced HuR by transfecting with a small interfering RNA against HuR (siHuR). Western blot analysis of ARID1A levels revealed that downregulation of HuR decreased ARID1A protein levels in both cell lines, indicating that HuR positively regulates ARID1A expression (Figure 2A,B). Given that HuR regulates the turnover and translation of target mRNAs [4], we evaluated *ARID1A* mRNA levels through RT-qPCR analysis after silencing HuR. As seen in Figure 2C,D, downregulation of HuR significantly decreased *ARID1A* mRNA levels in MDA-MB-231 and Hs578t cells, showing that HuR regulates *ARID1A* at the mRNA level. We also observed that this regulation may occur independently of irradiation, as no further suppression in the levels of ARID1A was seen in irradiated cells (Figure 2A,B).

To further confirm these findings, we used a pharmacological inhibitor of HuR, CMLD-2, a small molecule that abolishes the interaction between HuR and target mRNAs by competitive binding [43]. Consistent with our previous results, pharmacological inhibition of HuR led to a decrease in ARID1A in MDA-MB-231 and the SUM159PT cell lines (Figure 2E,F). These results further support our findings that HuR regulates expression of ARID1A by promoting the stability of its mRNA levels. They also show that this process requires the ability of HuR to bind its targets (Figure 2E,F). As a control, to verify the inhibition of HuR by CMLD-2, we evaluated the protein levels of a well-established HuR target, p27kip1 (Figure 2E,F) [44]. Consistent with an inhibitory role of HuR protein in p27 translation, CMLD-2 treatment induced endogenous p27 protein levels in both MDA-MB-231 and SUM159PT cells (Figure 2E,F). 

**Figure 3:**
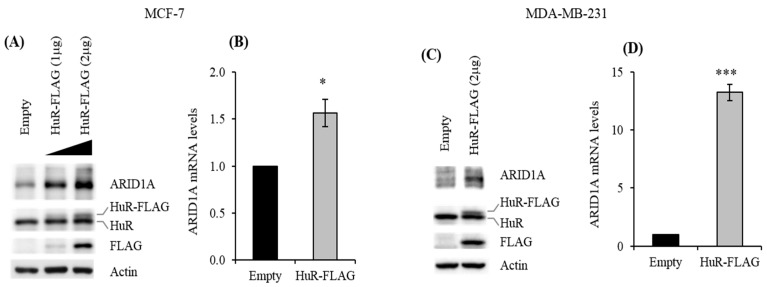
HuR overexpression elevates ARID1A abundance. MCF-7 and MDA-MB-231 cells transfected with an empty or a HuR-FLAG tagged vector were collected to evaluate HuR and ARID1A expression by Western blot (**A**,**C**) and RT-qPCR (**B**,**D**) analyses. Data represent the average of three independent experiments. Error bars represent SEM (standard error of the mean), * *p* ≤ 0.01 and *** *p* ≤ 0.0001.

To complement our studies and further validate the interaction between HuR and ARID1A, we evaluated the effect of HuR overexpression on ARID1A. For this purpose, we transiently overexpressed HuR using a FLAG-tagged HuR expression vector. We confirmed the overexpression of HuR by determining the expression of HuR and FLAG proteins (Figure 3A,C). Overexpression of HuR increased ARID1A protein and mRNA in both MCF-7 (Figure 3A,B) and MDA-MB-231 (Figure 3B,D) cells. While the increase in *ARID1A* mRNA in MDA-MBA-231 was greater than in MCF-7 following HuR overexpression, the expression level of ARID1A protein was observed to be much higher in MCF-7 cells. This difference in mRNA and protein expression of ARID1A is likely due to differential translation efficiency. However, the exact mechanism for the observed differences has not been studied and warrants further investigation. These results confirm once again that HuR positively regulates ARID1A expression. 

### 2.3. Correlation Between HuR and ARID1A Expression in Tumors of Patients with Breast Cancer

**Figure 4:**
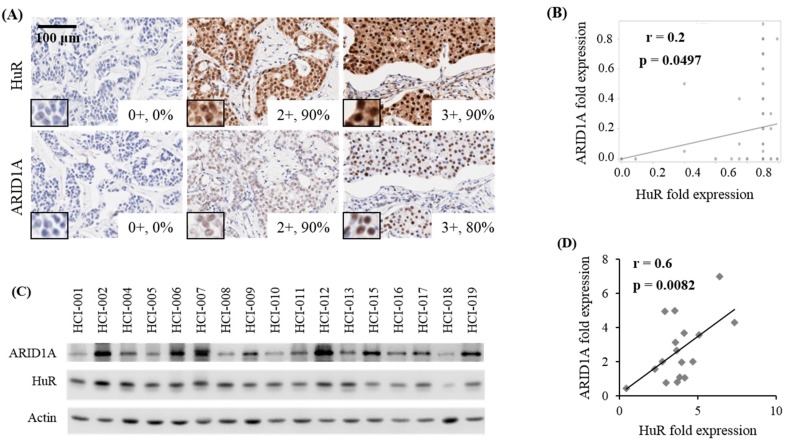
HuR and ARID1A expression levels correlate in vivo. (**A**) The levels of HuR and ARID1A in breast cancer were examined by immunohistochemical (IHC) analysis of tissue arrays. Scale bar equals 100 µm (**B**) Analysis of correlation in the levels of HuR and ARID1A in breast cancer tissue arrays. (**C**) Patient-derived xenograft tissues were analyzed by Western blot to evaluate HuR and ARID1A expression levels. (**D**) Quantified and normalized band intensities of ARID1A and HuR were subjected to correlation analysis.

Our in vitro data and the publicly available in silico data established that HuR positively regulates ARID1A expression. To validate our findings, we examined whether expression of these two proteins was associated in breast cancer tissues. We performed immunohistochemical (IHC) analysis of tumor microarrays (TMA) of 100 samples that included primary and metastatic tumors, as well as different tumor grades and stage subsets (Figure 4A). Nuclear expression of antibody was scored from 0–3 in tumor cells with the percentage of immunoreactive tumor cells quantified as a percentage. Semi-quantitative scores of ARID1A and HuR protein expression levels were subjected to correlation analyses to detect any co-expression between ARID1A and HuR in all patients or subtypes. No significant correlation was found when samples were analyzed by subtypes. However, when analyzing all patients with breast cancer, a moderate and significant correlation between ARID1A and HuR expression levels was observed (Figure 4A,B). We also evaluated the association between the expression of HuR and ARID1A proteins in vivo using a collection of patient-derived breast tumor xenografts (PDXs) by Western blot analysis (Figure 4C). PDXs are important in vivo models not only because they preserve the original biological characteristics of the patient tumor, but also because they are characterized as very aggressive tumors derived from patients who had a short survival time [45]. After quantifying band intensities and normalizing them to actin, we found a strong and significant correlation between the abundance of ARID1A and HuR, supporting our findings that HuR promotes ARID1A expression in breast cancer tumors (Figure 4C,D).

To obtain more evidence to support our findings, we analyzed other independent studies. A The Cancer Genome Atlas (TCGA) study shows that mRNA levels of HuR and ARID1A are found to be elevated in breast cancer patients. This study also shows that the expression levels of *HuR* and *ARID1A* mRNA correlate moderately (Supplementary Figure S2A–C) [46]. Likewise, the Stickeler study also shows that the levels of *HuR* and *ARID1A* mRNA are elevated in TNBC patients [47]. Nevertheless, the correlation between HuR and ARID1A expression, in this case, was strong (Supplementary Figure S2D–F).

Due to these observations, we next wanted to evaluate whether there was a clinical relevance of HuR and ARID1A expressions in breast cancer patients. For this, we performed disease-free survival curve analysis on breast cancer patients expressing high levels of both HuR and ARID1A. We found that the probability of disease-free survival was severely reduced in patients expressing high levels of HuR and ARID1A, highlighting the clinical role of the HuR–ARID1A axis in patients with breast cancer (Supplementary Figure S2G). 

### 2.4. Genetic Inhibition of ARID1A Radiosensitizes Breast Cancer Cell Lines

**Figure 5:**
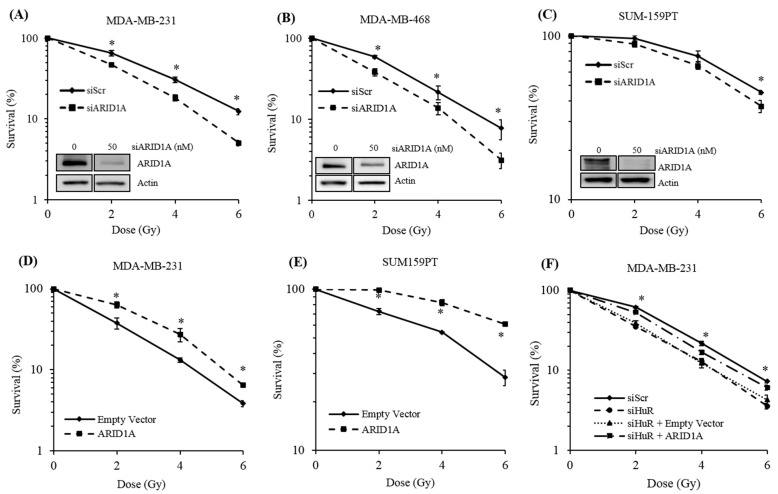
Targeting ARID1A sensitizes breast cancer cells to radiation. (**A**–**C**) MDA-MB-231, MDA-MB-468, and SUM159PT cells transfected with ARID1A siRNA were exposed to the indicated doses of gamma radiation and re-plated for colony formation. Around 10 days later, the colonies were stained and quantitated to evaluate surviving fractions. (**D**,**E**) MDA-MB-231 and SUM-159PT cells transfected with either an empty vector or an ARID1A overexpressing vector were irradiated with the indicated doses and re-plated for colony formation analysis. Around 10 days later, the colonies were stained and quantitated to evaluate the surviving fractions. (**F**) MDA-MB-231 cells previously treated with HuR siRNA were transfected with ARID1A or empty vector to test the ability of ARID1A to complement HuR-dependent radioresistance. Error bars represent SEM (standard error of the mean), * *p* ≤ 0.05.

In a recent study, we showed that by controlling the DNA damage response, HuR plays a significant role in the resistance of TNBC cells to gamma radiation [18]. Here, we show that HuR promotes the expression of ARID1A by stabilizing the *ARID1A* mRNA. To assess a potential role of ARID1A in the radiation resistance conferred by HuR, we downregulated ARID1A in a panel of human breast cancer cell lines—MDA-MB-231, MDA-MB-468, and SUM159PT—and assessed their clonogenic survival potential. ARID1A silencing significantly sensitized all three cell lines to the cytotoxic effects of radiation compared to control siRNA transfected cells (Figure 5A–C). To confirm these observations, we calculated the dose enhancement factor (DEF) for these assays. A DEF higher than one indicates that the treatment has a radiosensitizing effect, whereas DEF lower than one indicates that the treatment is radioprotective. We obtained DEF values of 1.3, 1.25, and 1.16 for MDA-MB-231, MDA-MB-468, and SUM159PT cells treated with ARID1A siRNA, respectively. These DEF values indicate that inhibition of ARID1A confers radiosensitization to the breast cancer cells. Conversely, exogenous overexpression of ARID1A increased radioresistance when compared with control cells expressing an empty vector (Figure 5D,E). Altogether, these results further imply that ARID1A plays an important role in radioresistance in breast cancer cells via the HuR–ARID1A axis.

### 2.5. ARID1A Expression Complements HuR Inhibition During Resistance to Radiation

We have previously shown that genetic inhibition of HuR in TNBC cells enhances radiosensitivity [18]. This raises the question of whether ARID1A could rescue HuR ablation mediated radiosensitivity in TNBC cells. To test this possibility, MDA-MB-231 cells in which HuR was genetically silenced using an siRNA were transfected with either an empty vector or an expression vector carrying the coding region of ARID1A. These cells were then subjected to clonogenic assays. While empty vector showed no effect, expression of ARID1A rescued the clonogenic survival of cells in which HuR was downregulated. The DEF value in siHuR-treated cells changed from 1.23 to 0.86 following ARID1A overexpression, suggesting that ARID1A is a major effector of the resistance to radiation conferred by HuR (Figure 5F).

### 2.6. Impact of ARID1A on Radiation-Induced DSB in HuR-Inhibited Cells

**Figure 6:**
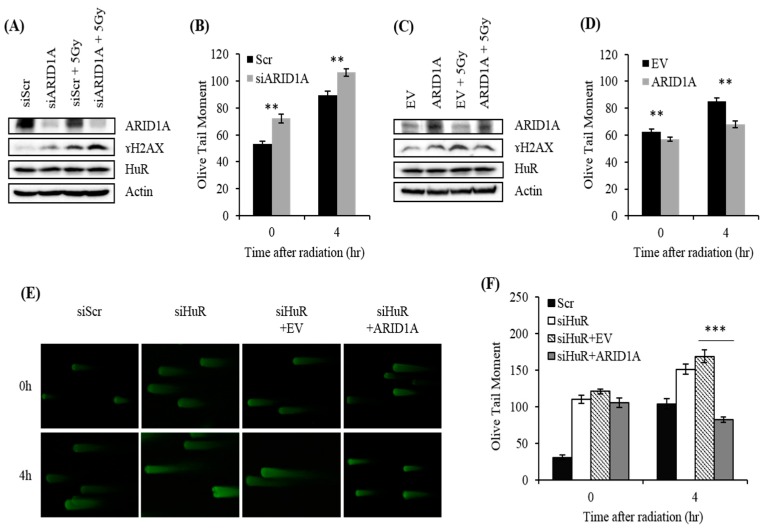
Ablating ARID1A leads to accumulation of DNA double-strand breaks (DSBs). In MDA-MB-231 cells transfected with ARID1A siRNA, followed by irradiation, the levels of DNA damage were evaluated with two approaches: Measurement of ɤH2AX levels by (**A**) Western blot analysis and (**B**) neutral comet assay. After MDA-MB-231 cells transfected with an empty vector or one that overexpresses ARID1A were irradiated, the levels of DNA damage were evaluated with two approaches: Measurement of ɤH2AX levels by Western blot analysis (**C**) and neutral comet assay (**D**). (**E**,**F**) MDA-MB-231 cells previously treated with HuR siRNA were transfected with an empty vector or a vector that expressed ARID1A to test the ability of ARID1A to reduce DNA DSBs in cells where HuR was downregulated. Error bars represent SEM (standard error of the mean), ** *p* ≤ 0.05; *** *p* ≤ 0.0001.

We previously reported that HuR contributes to radioresistance mainly by engaging the DNA damage response pathway [18]. To further explore the role of ARID1A in radiation resistance conferred by HuR, we evaluated the impact of ARID1A expression on radiation-induced DSBs. Compared with their control, cells in which ARID1A was silenced demonstrated a slight increase in ɤH2AX levels, possibly due to the role of ARID1A in promoting DNA repair of spontaneous DSBs (Figure 6A). However, cells in which ARID1A was silenced demonstrated higher accumulation of DSBs following a 2 h treatment with gamma radiation, as indicated by Western blot analysis of ɤH2AX levels and neutral comet assays (Figure 6A,B). Conversely, ectopic overexpression of ARID1A reduced the levels of radiation-induced DSBs, suggesting that ARID1A plays an important role in the resistance to radiation by suppressing the accumulation of DSBs (Figure 6C,D). It is important to note that overexpression of ARID1A did not influence HuR expression levels (Figure 6A,C). Finally, we evaluated the ability of ARID1A overexpression to reduce radiation-induced DSBs in cells where HuR was inhibited. Compared with control cells, genetic inhibition of HuR led to a higher accumulation of DSBs caused by radiation, as evidenced by the formation of longer comet tails (Figure 6E,F) [18]. While empty vector had no effect on the levels of DSBs, forced expression of ARID1A drastically reduced DSBs after radiation, further demonstrating that ARID1A plays an important role in HuR-dependent resistance to radiation (Figure 6E,F).

## 3. Discussion

In the present study, we investigated the ability of HuR to modulate ARID1A expression in breast cancer and its contribution to radioresistance. We show that HuR and *ARID1A* mRNA associate with each other and that HuR acts as a novel post-transcriptional regulator of ARID1A expression. We found that HuR binds to the distal portion of the *ARID1A* 3′-UTR and this interaction allows HuR to enhance ARID1A expression by extending the half-life of the *ARID1A* mRNA. 

Our findings are consistent with other studies demonstrating that HuR plays general roles as an mRNA stabilizer by binding to specific ARE motifs in its mRNA targets, such as those encoding c-Fos, c-Myc, VEGF, cyclooxygenase-2, p21, p53, NPM, XIAP, ATF2, and MKP-1 [48,49,50,51,52]. We found that HuR and ARID1A expression levels are positively correlated in samples of breast tumors and patient-derived xenografts, as well as in other independent studies. We also found that this association has potentially important implications in tumor biology in breast cancer. Our results show that HuR-mediated expression of ARID1A presents a great potential as a therapeutic target. In fact, increased levels of endogenous ARID1A caused by HuR contribute to increased resistance to radiation. We observed by comet assays and levels of γH2AX that ARID1A expression reduces radiation-induced DNA fragmentation. These results are consistent with a previously published study, where ARID1A promotes NHEJ DNA repair, conferring resistance to UV, ionizing radiation, and cisplatin in lung and bone osteosarcoma cells [28].

In summary, we demonstrate for the first time that HuR binds to the *ARID1A* 3′-UTR and regulates its mRNA stability in TNBC cells. We also observed this regulation in non-TNBC cells such as MCF-7 and in non-small cell lung cancer (NSCLC) cells (data not shown). HuR-induced expression of ARID1A is essential to reduce the levels of DNA fragmentation during radiation, expanding our understanding of the mechanisms by which HuR promotes radioresistance in breast cancer. In addition, the fact that HuR–*ARID1A* interaction contributes to radiation resistance points that ARID1A can play roles as a tumor promoter even though it is mainly described as a tumor suppressor. It also highlights the importance of targeting the HuR–ARID1A axis in order to sensitize cancer cells to chemotherapy and radiation.

## 4. Materials and Methods

### 4.1. Cell Culture

MDA-MB-231, Hs578t, MCF-7, and MDA-MB-468 cells were purchased from American Type Culture Collection (ATCC). MDA-MB-231 and MCF-7 were cultured in Dulbecco’s Modified Eagle Medium (DMEM) (Corning CellGro., Manassas, VA, USA) supplemented with 10% fetal bovine serum (Sigma-Aldrich, St. Louis, MO, USA), 1% pen–strep, and 1% L-glutamine (Corning, CellGro). MDA-MB-468 was cultured in DMEM (Corning CellGro) supplemented with 10% fetal bovine serum, 1% pen–strep, 1% L-glutamine, 1% sodium pyruvate, and 1% Minimum Essential Medium (MEM) amino acids (Corning CellGro). Hs578t was cultured in MEM supplemented with 10% fetal bovine serum, 1% pen–strep, and 1% L-glutamine (Corning CellGro). SUM159PT cells were obtained from Asterand and cultured in Ham’s/F-12 (Corning CellGro) supplemented with 10% fetal bovine serum, 1% pen–strep, 1% L-glutamine, 10 mM 4-(2-hydroxyethyl)-1-piperazineethanesulfonic acid (HEPES), 1 μg/mL hydrocortisone, and 5 μg/mL insulin. All cell lines were maintained in a humidified incubator at 5% CO_2_ and 37 °C. 

### 4.2. Plasmids and Reagents

HuR–FLAG was a kind gift from Dr. Myriam Gorospe. ARID1A-V5 was obtained from the nonprofit plasmid repository Addgene [53]. pLenti-Luciferase-ARID1A 3′UTR was purchased from Applied Biological Materials, Inc. (ABM, Richmond, BC, Canada). Actinomycin D (Sigma) was dissolved in Dimethyl sulfoxide (DMSO) and used at the final concentration of 5 μg/mL. The HuR inhibitor, CMLD-2, was synthesized and used as previously described [43,54]. The compound was dissolved in DMSO and used in the studies described herein. DMSO without the compound was used as a control.

### 4.3. PDX Tissue Samples and Tissue Arrays

Breast cancer patient-derived xenograft (PDX) tissues for our analysis were provided by Dr. Alana Welm. PDX mice were produced and maintained as previously described [45,55]. Breast cancer tissue arrays were obtained from US Biomax, Inc. (Rockville, MD, USA). The analyses of stained sections were performed by a qualified and experienced pathologist and statistician. Briefly, immunohistochemical stains were assessed by qualitative scoring on a scale of 0–3 with 0 as negative, 1+ as weak, 2+ as moderate, and 3+ as strong nuclear staining. The percentage of immunoreactive nuclei was assessed and quantified on a range of 0–100% of tumor cells staining.

### 4.4. Transient Transfection

Cells were transfected with 20 nM of HuR siRNA (Dharmacon, Lafayette, CO, USA) or 50 nM of ARID1A siRNA (Santa Cruz, Dallas, TX, USA) using the DharmaFECT2 transfection reagent (Dharmacon), following the manufacturer’s instructions. MDA-MB-231 and Hs578t cells (1 × 10^5^) were seeded in 6-well plates and transfected with 20 nM of HuR siRNA or 50 nM ARID1A siRNA (Dharmacon) using DharmaFECT2 transfection reagent (Dharmacon), as previously described [18]. Six hours after transfection, the medium was replaced with MEM media containing 2% serum. Cells transfected with scrambled siRNA served as controls; 24 h later, the cells were harvested and total cell lysates were prepared for mRNA and protein expression analysis. 

### 4.5. RT-qPCR

Reverse transcription (RT) followed by real-time quantitative (q)PCR analysis was performed as previously described [18]. Briefly, total RNA from siScr-, siHuR-, and siARID1A-treated MDA-MB-231, Hs578t, and MCF-7 cells was isolated using TRIZOL reagent (Life Technologies, Carlsbad, CA, USA), following the manufacturer’s protocol. cDNA obtained by reverse transcription using Omniscript Reverse Transcription Kit (Qiagen, Inc., Germantown, MD, USA) was used to perform quantitative real time PCR (qRT-PCR) using PerfeCTa SYBR Green Fast Mix (Quanta Biosciences, Beverly, MA, USA) and the following set of oligonucleotide primers for detection of *ARID1A* (5′-CCT GAA GAA CTC GAA CGG GA-3′ and 5′-CGG CTC CGT GAG GTT ATT G-3′), *HuR* (5′-ATG AAG ACC ACA TGG CCG AAG ACT-3′ and 5′-AGT TCA CAA AGC CAT AGC CCA AGC-3′), and *GAPDH* (5′-AGC CTC AAG ATC AGC AAT GCC-3′ and 5′-TGT GGT CAT GAG TCC TTC CAC GAT-3′). Using GAPDH as internal control, the expression abundance of targeted genes was determined in triplicate using the ΔΔCt method. Experiments were conducted at two separate times, and the data obtained were analyzed for statistical significance. Data shown are average of the two experiments.

### 4.6. Western Blotting

Cells were lysed with RIPA buffer (Thermo Scientific, Waltham, MA, USA) supplemented with Halt Protease Inhibitor Cocktail and Halt Phosphatase Inhibitor Cocktail (Thermo Scientific). Equal volumes of 30 μg of whole cell lysate and 2X Laemmli buffer were mixed, boiled for 5 min, and resolved on 10% polyacrylamide gels. After transfer, membranes blocked with 5% nonfat dry milk were incubated with antibodies against HuR (1:1000, clone 3A2 Santa Cruz), p27 (1:5000 Santa Cruz), actin (1: 5000 Millipore, Danvers, MA, USA), FLAG (anti DYKDDDDK Tag, 1:1000 Cell Signaling, Danvers, MA, USA), γ-H2AX (1:1000 Cell Signaling), or ARID1A (1:1000 Cell Signaling). 

### 4.7. Ribonucleoprotein Immunoprecipitation (RIP) Analysis

MDA-MB-231 cells, plated at 50% confluence on 10 cm plates, were harvested 24 h later and subjected to immunoprecipitation using either 30 μg of anti HuR antibody (clone 3A2 Santa Cruz) or immunoglobulin (IgG) control (Santa Cruz), as indicated by Keene et al. [56]. RNA isolated from either immunoprecipitation or 10% input were subjected to qRT-PCR analysis. RNA fold enrichment to input was calculated and normalized to IgG and H2AX negative control.

### 4.8. Luciferase Assay

MDA-MB-231 cells (100,000) plated on 6-well plates were transfected with control (siScr) or HuR-directed (siHuR) siRNAs. Twenty-four hours later, cells were subjected to a second transfection with 100 ng of the corresponding luciferase vectors. Sixteen hours later, lysates were collected and analyzed for luciferase activity in a luminometer using the Dual-Luciferase^®^ Reporter Assay System (Promega Corporation, Madison, WI, USA), according to the manufacturer’s instructions. A Renilla luciferase vector was used as a normalizing control.

### 4.9. Analysis of ARID1A mRNA Stability in HuR-Silenced Cells

The stability of *ARID1A* mRNA was assessed in MDA-MB-231 cells transfected with either siScr or siHuR and treated 24 h later with actinomycin D (5 µg/mL) in the cell culture medium to inhibit mRNA transcription. At the indicated time points, the relative amount of specific mRNA remaining in each sample could be correlated with mRNA degradation. Total RNA was prepared as described [18]. Total RNA was extracted at 0, 2, 4, 6, and 8 h after actinomycin D treatment, and the endogenous *ARID1A* mRNA levels were analyzed by RT-qPCR. Since the mRNA levels for *H2AX* were unchanged after actinomycin D treatment, *H2AX* was used as a reference gene. *ARID1A* mRNA half-lives were calculated from typical decay curves by linear regression between 0 and 8 h [57]. Values ± standard deviation (SD) are based on at least two independent experiments.

### 4.10. Exposure to Ionizing Radiation

Cells were exposed to radiation using a Gamma Cell 40 Exactor (Best Theratronics, Ottawa, ON, Canada) at a dose rate of 0.81 Gy/min at room temperature. Immediately after exposure, the cells were returned to the incubator and harvested at specified time points for each experiment. 

### 4.11. Clonogenic Assay

MDA-MB-231, MDA-MB-468, and SUM-159PT cells were transfected with 100 nM siHuR, 50 nM ARID1A siRNA, or control siRNA (siScr), and irradiated at 2, 4, and 6 Gy using a Gammacell 40 Extractor (Nordion International, Ottawa, ON, Canada) 24 h later. Cells were then trypsinized, plated in triplicate in two sets of different cell numbers, and incubated for 10–14 days to allow colony formation. Once colonies reached a given size, the plates were stained with 0.5% gentian violet in methanol. Colonies were counted once they reached the cut-off of 50 cells per colony. 

### 4.12. Biotinylated RNA Pulldown

DNA sequences corresponding to the *ARID1A* 3′UTR (full length, 1–829 and 830–1354) were cloned into pGEM T-Easy vectors (Promega) and linearized by digestion with MluI (New England Biolabs, Ipswich, MA, USA). For positive and negative controls, we respectively used 3′UTRs of c-Myc and GAPDH previously cloned into pGEM T-Easy vectors, a kind donation of Dr. Anita Corbett from the Department of Biochemistry at Emory University, Atlanta, GA [58]. Biotinylated RNA segments were generated by in vitro transcription using the T7 Maxiscript kit (Ambion, Austin, TX, USA) with biotin-11-cytidine-5-triphosphate (biotin-11-CTP; Roche Applied Science, Indianapolis, IN, USA). The biotinylated probes were incubated with MDA-MB-231 whole-cell lysates and recovered with neutravidin-coated beads (Thermo Scientific) for Western blot analysis.

### 4.13. Data Mining

*ARID1A* and *HuR* mRNA expression levels were analyzed in normal breast and breast cancer patient samples using the following cohorts from Oncomine and cBioportal: The Cancer Genome Atlas (TCGA) (*n* = 593, *n* = 1084) and Stickeler et al. (*n* = 57) [47]. Correlation analyses with *ARID1A* and *HuR* mRNA expression were carried out from malignant samples. *T*-test was used to determine significant differences in *ARID1A* and *HuR* expression levels in different patient populations. Time to outcome analyses (disease-free survival duration) were examined using Kaplan–Meier and statistical significant differences were determined using the log-rank test, and the *ARID1A/HuR* high patient population was separated using a threshold of Z score + 1 for both genes.

### 4.14. Comet Assay

The ability of ARID1A to influence DNA DSBs either alone or in the context of HuR knockdown in the presence and absence of radiation was determined using the neutral comet assay (Comet Assay Kit, Trevigen, Gaithersburg, MD, USA), as described previously [18]. Cells were seeded in 35-mm dishes and allowed to attach for 24 h. Cells were then exposed to the appropriate treatment and subsequently irradiated with single dose of 20 Gy. Immediately and 4 h after irradiation, cells were harvested, washed with ice-cold phosphate buffered saline (PBS), and resuspended in 1% low-melting agarose. The agarose/cell mixture was spread onto glass slides and the slides were immersed in ice-cold neutral lysis buffer at 4 °C overnight. After lysis, the slides were electrophoresed in 1X TBE buffer at 4 °C for 45 min, fixed with 70% ethanol and stained with SYBR Green for 30 min in the dark. Comet images were captured using a Nikon microscope and olive tail moment was measured for at least 50 comets per sample using the Casplab comet assay software. 

## 5. Conclusions

This study identifies *ARID1A* as a novel HuR target and provides evidence for a pivotal role of the HuR/ARID1A axis in breast cancer radioresistance, thereby supporting the potential therapeutic targeting to enhance radiotherapy in breast cancer.

## Supplementary Materials

The following are available online at https://www.mdpi.com/2072-6694/11/12/2014/s1, Figure S1: HuR binding sites on ARID1A 3’UTR, Figure S2: HuR and ARID1A expression levels correlate in vivo.
