# Supplementary material for: HuR Reduces Radiation-Induced DNA Damage by Enhancing Expression of ARID1A
Source: Cancers (Basel). 2019 Dec 13;11(12):2014. doi: 10.3390/cancers11122014 (PMC6966656; doi:10.3390/cancers11122014)
Supplement: Supplementary file 1 [file cancers-11-02014-s001.zip › cancers-621202-supplementary-final.pdf]

cancers

Article

HuR Reduces Radiation-Induced DNA Damage by Enhancing Expression of ARID1A

Daniel Andrade, Meghna Mehta, James Griffith [1], Sangphil Oh, Joshua Corbin [4], Anish Babu, Supriyo De, Allshine Chen, Yan D. Zhao, Sanam Husain, Sudeshna Roy, Liang Xu, Jeffrey Aube, Ralf Janknecht, Myriam Gorospe, Terence Herman, Ramesh Rajagopal and Anupama Munshi

Supplementary Materials

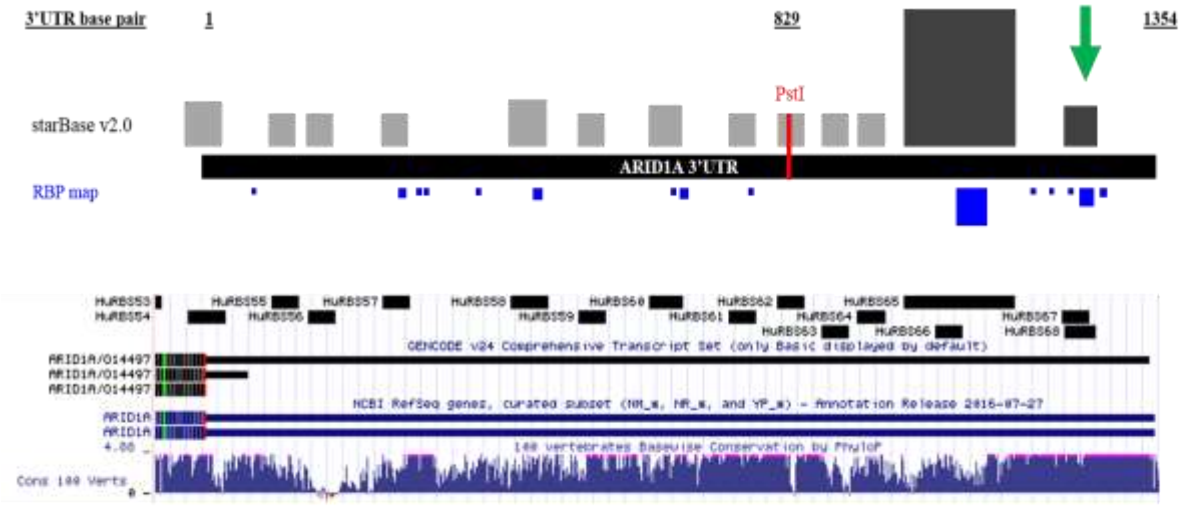

Figure 1. HuR binding sites on ARID1A 3′UTR. ARID1A 3′UTR region showing potential HuR-binding sites by two different online tools (starBase and RBP map). It also shows the relative position of PstI cleavage that delimits segment-1 and segment-2. The arrow shows a region on ARID1A 3′UTR that contains common predicted HuR binding sites that are also highly conserved in 100 species.

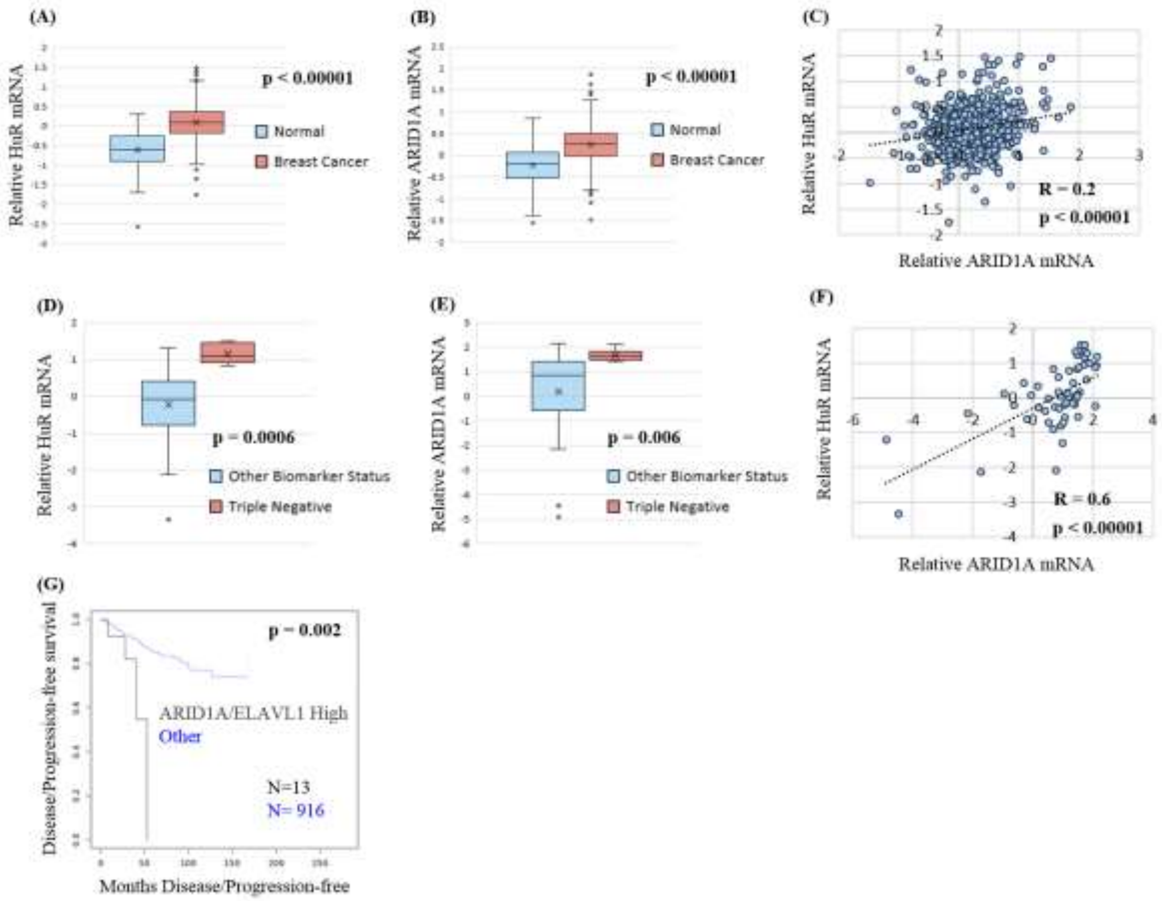

Figure 2. HuR and ARID1A expression levels correlate *in vivo*. (**A-C**) TCGA study shows elevated levels of HuR mRNA (A_23_P388681) and ARID1A mRNA (A_24_P92951) with moderate correlation in breast cancer patients. (**D-F**) Stickeler study shows elevated levels of HuR mRNA (A_23_P388681) and ARID1A mRNA (A_24_P92951) with a strong correlation in TNBC patients. (**G**) Disease free survival curve of breast cancer patients expressing high levels of both HuR and ARID1A.
