# Supplementary material for: HuR Reduces Radiation-Induced DNA Damage by Enhancing Expression of ARID1A
Source: Cancers (Basel). 2019 Dec 13;11(12):2014. doi: 10.3390/cancers11122014 (PMC6966656; doi:10.3390/cancers11122014)
Supplement: Supplementary file 1 [file cancers-11-02014-s001.zip › cancers-521202-supplementary-westerns blot figures-final.pdf]

Fig 1 C

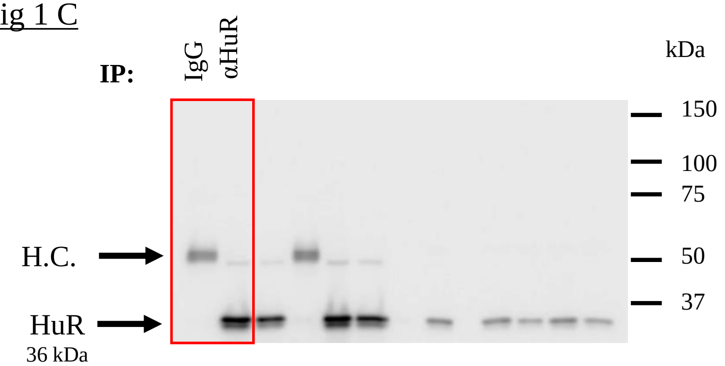

Fig 1 F

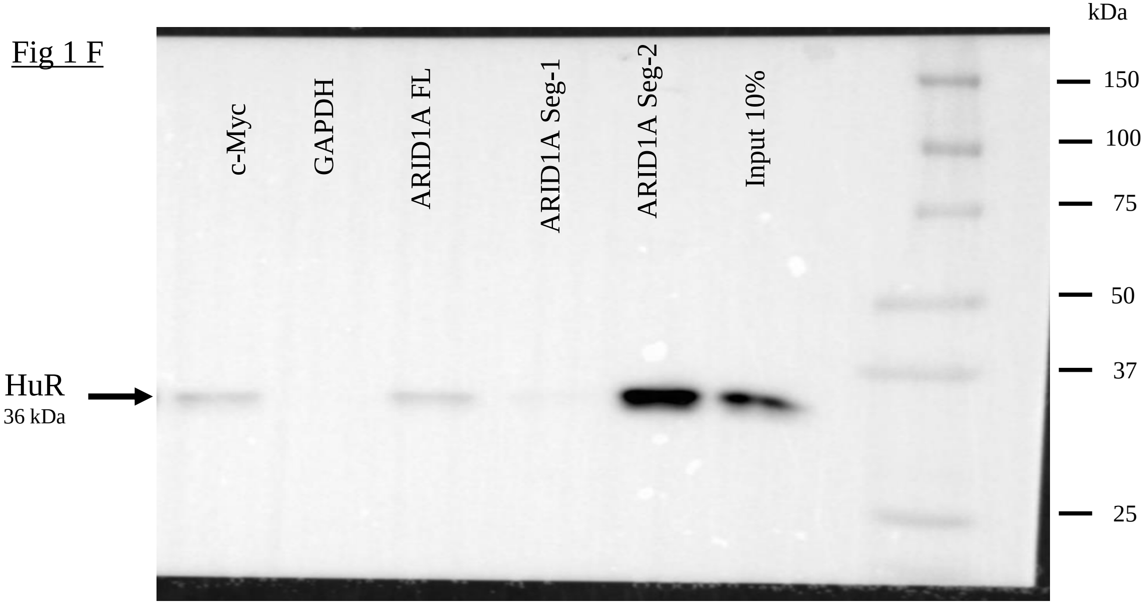

c-myc	GAPDH	ARID1A FL	ARID1A Seg-1	ARID1A Seg-2	Input
1.2	0	1	0.1	4.7	3.8

Fig 2 A

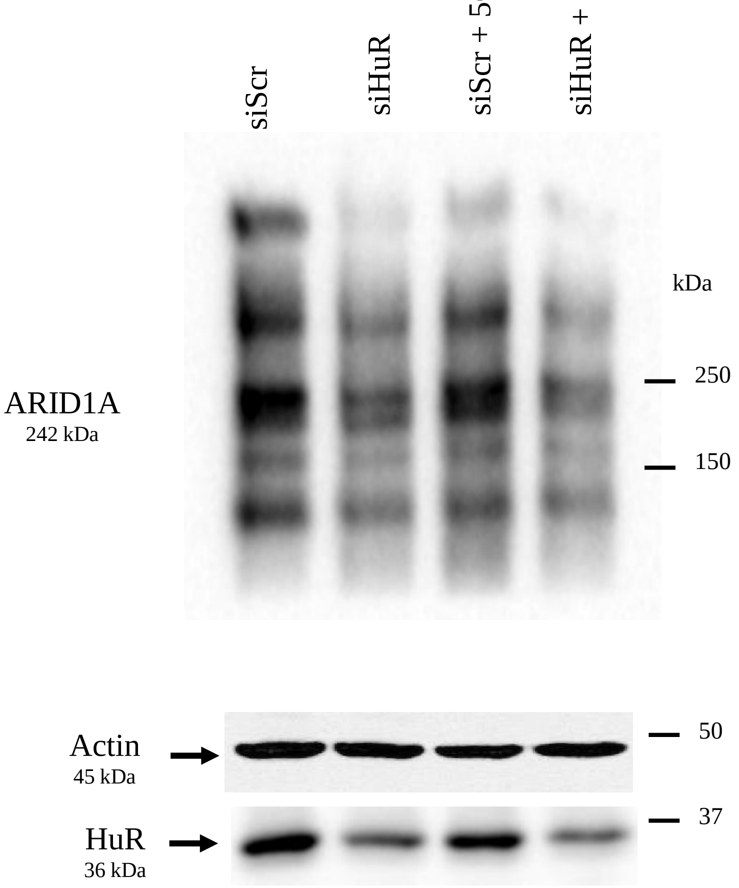

	siScr	siHuR	siScr+5Gy	siHuR+5Gy
ARID1A	1	0.7	1	0.5
HuR	1	0.6	0.9	0.6
Actin	1	1	1	1

Fig 2 B

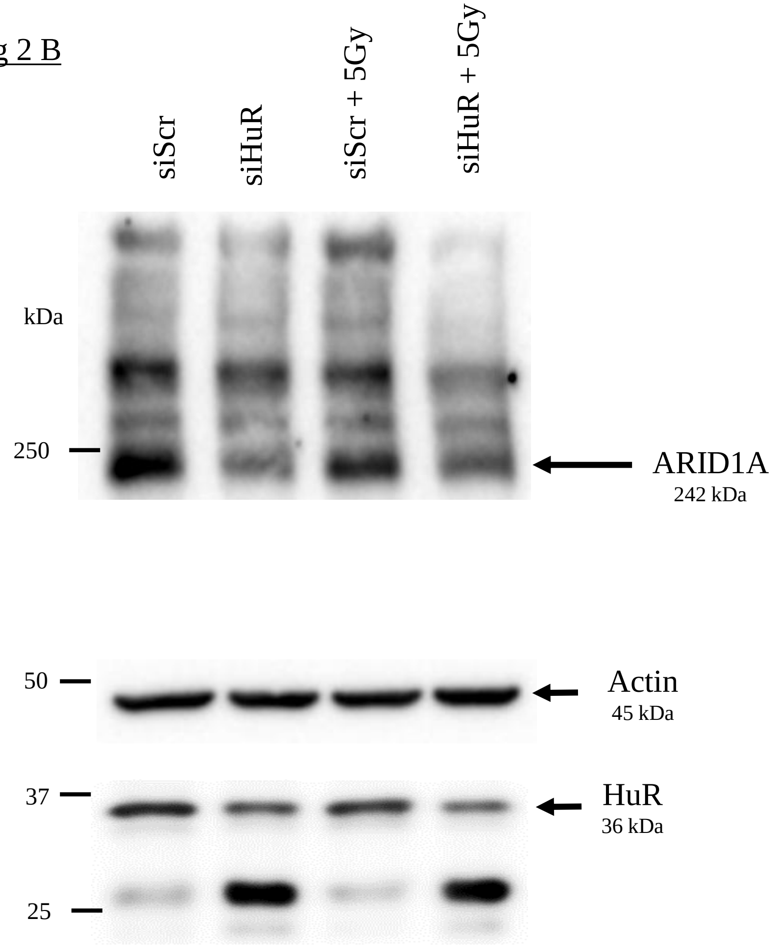

	siScr	siHuR	siScr+5Gy	siHuR+5Gy
ARID1A	1	0.6	1.1	0.7
HuR	1	0.7	1	0.5
Actin	1	1	1	1

Fig 2 E

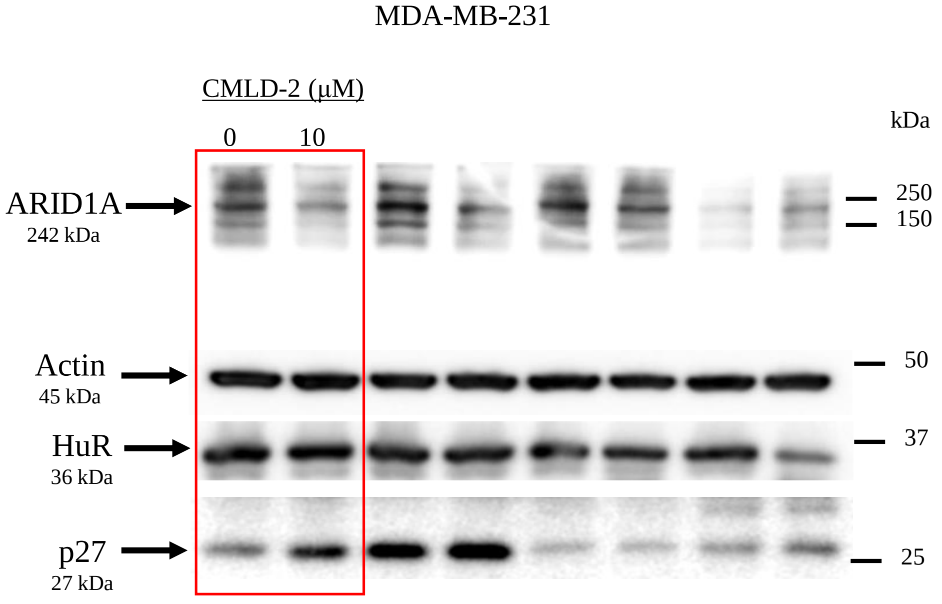

	0 uM	10 uM
ARID1A	1	0.6
HuR	1	1
p27	1	1.7
Actin	1	1

Fig 2 F

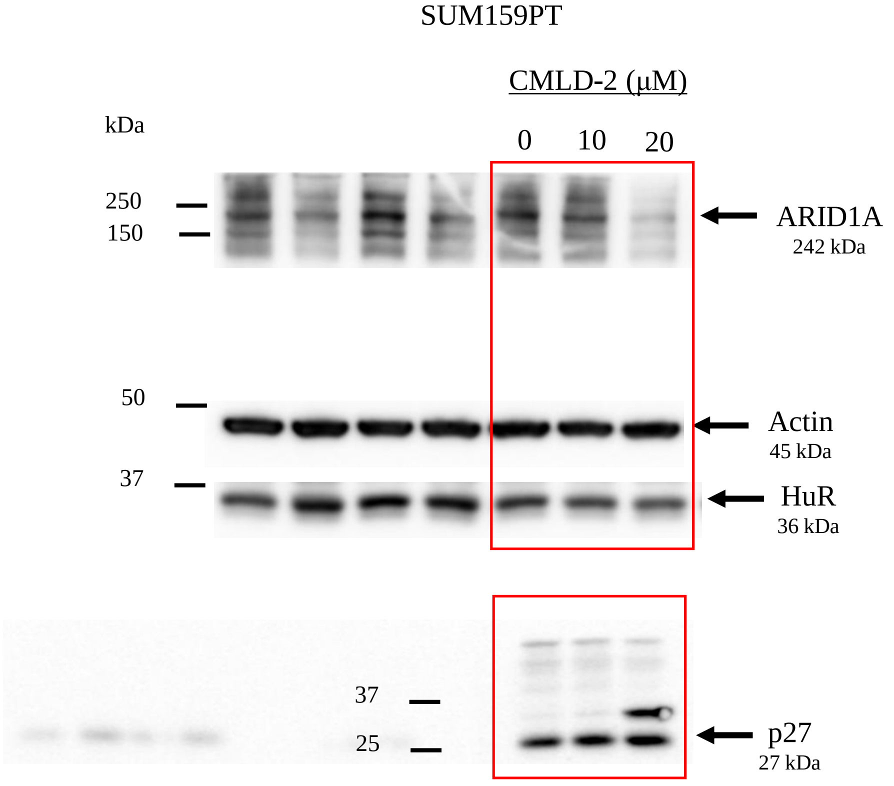

	**0**	**10**	**20**
ARID1A	1	1.1	0.3
HuR	1	1	0.9
p27	1	1.5	1.6
Actin	1	1	1

Fig 3A

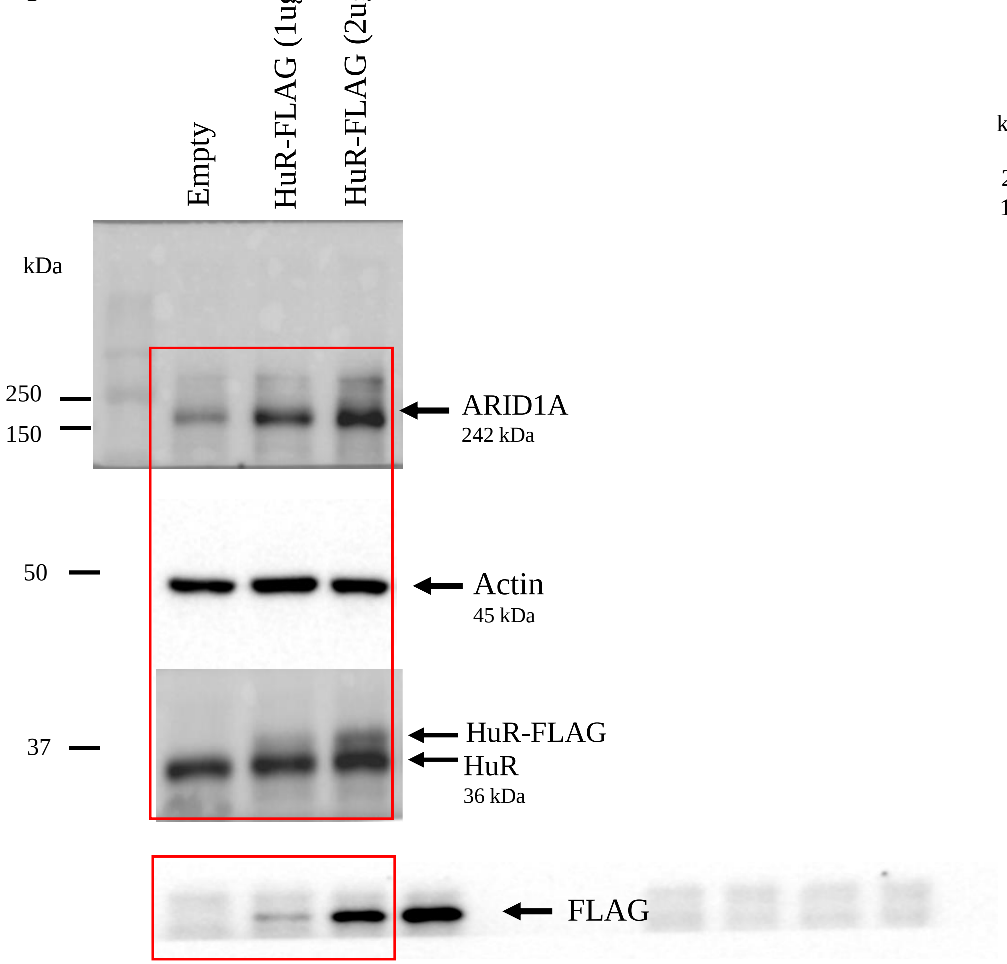

ARID1A	1	1.8	2.2
HuR	1	1.2	1.5
FLAG	0	0.9	3.7
Actin	1	1	1

Fig 3C

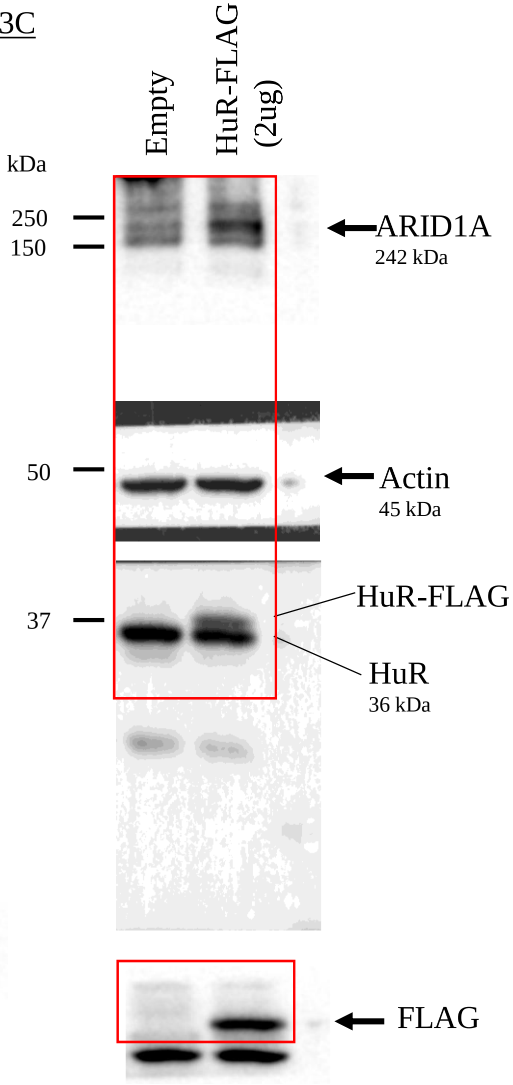

ARID1A	1	4.9
HuR	1	1.2
Actin	1	1

Fig 4C

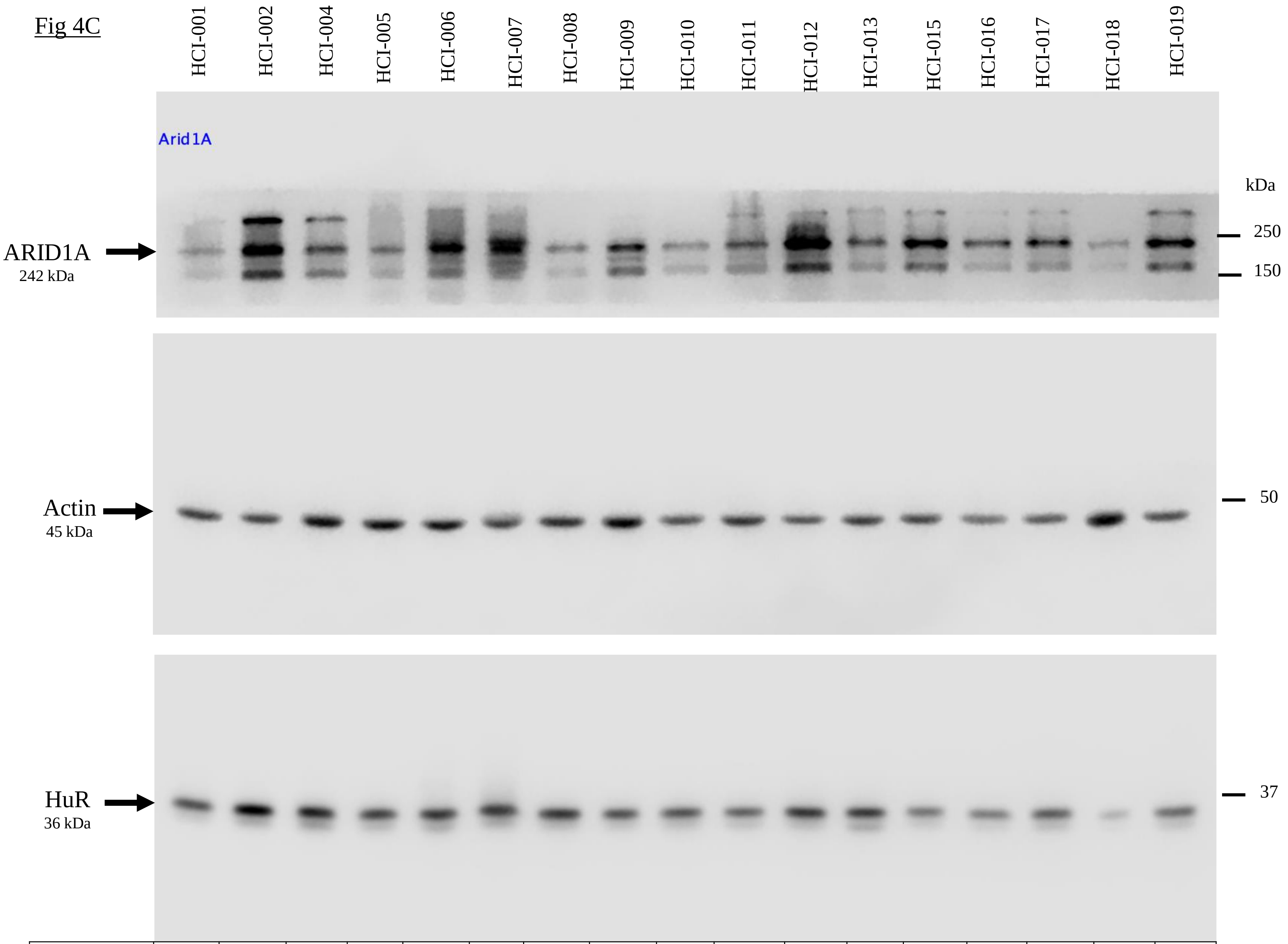

ARID1A	1.1	4.3	2	0.8	2.7	3.6	0.8	1.6	1	2	7	2	4.9	3.1	3.7	0.4	5
HuR	3.8	7.3	4	3	3.7	5.1	3.7	2.3	4.2	2.7	6.4	4.7	2.9	3.6	4.1	0.4	3.6
Actin	1	1	1	1	1	1	1	1	1	1	1	1	1	1	1	1	1

Fig 5A Fig 5B

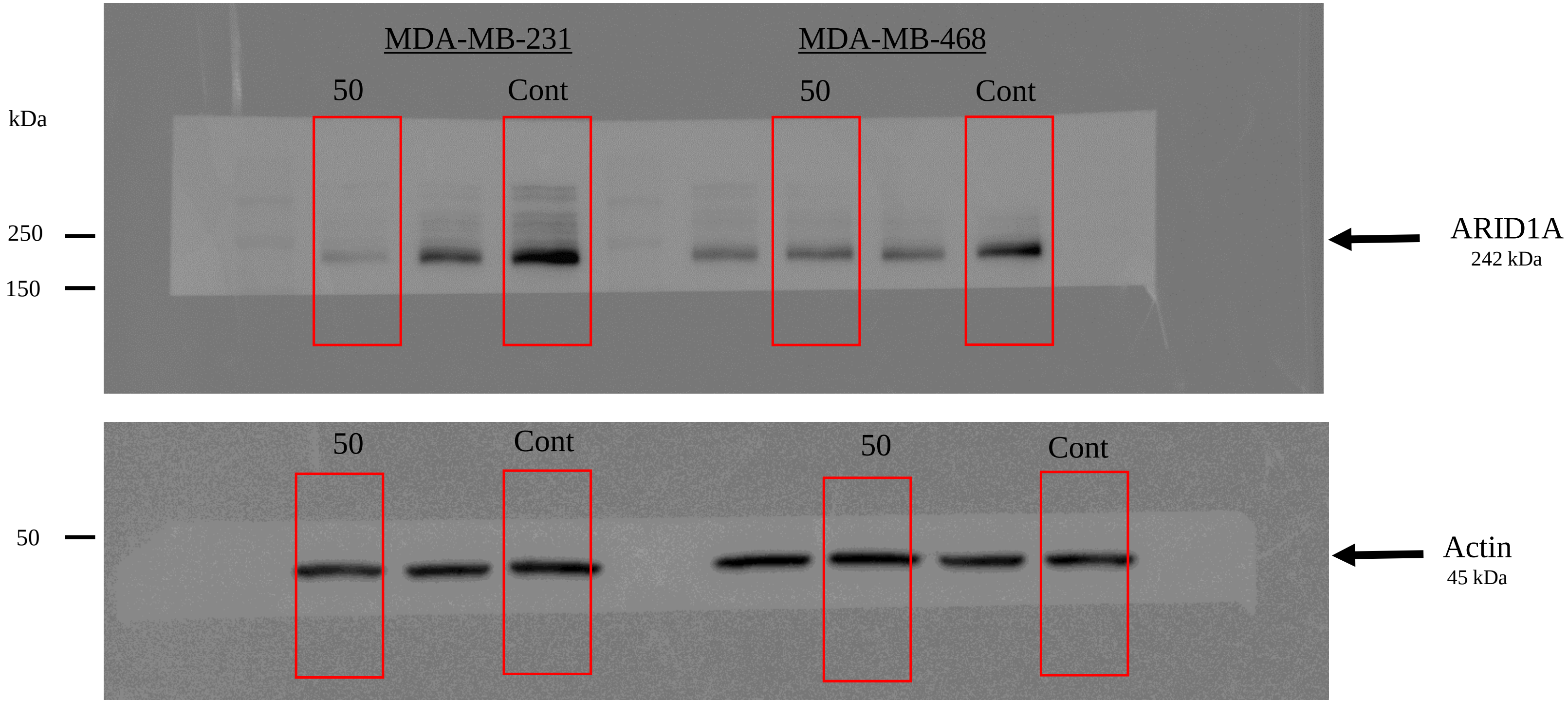

	**50**		**Cont**
ARID1A	0.2		1
Actin	1		1

	**50**		**Cont**
ARID1A	0.5		1
Actin	1		1

Fig 5C

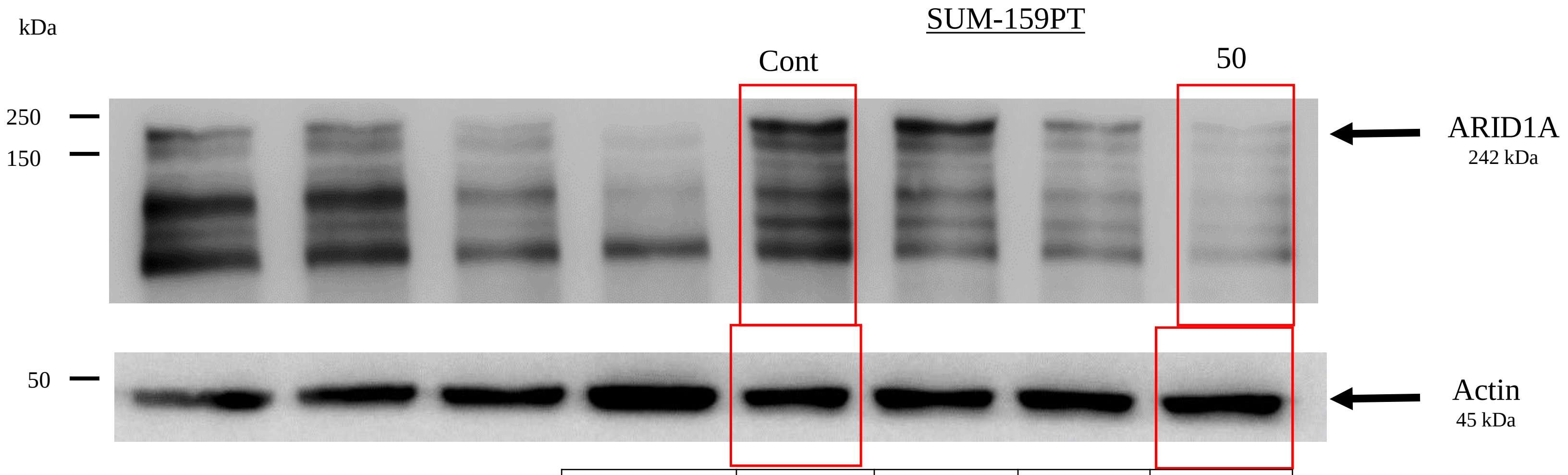

	Cont			50
ARID1A	1			0.1
Actin	1			1

Fig 6A

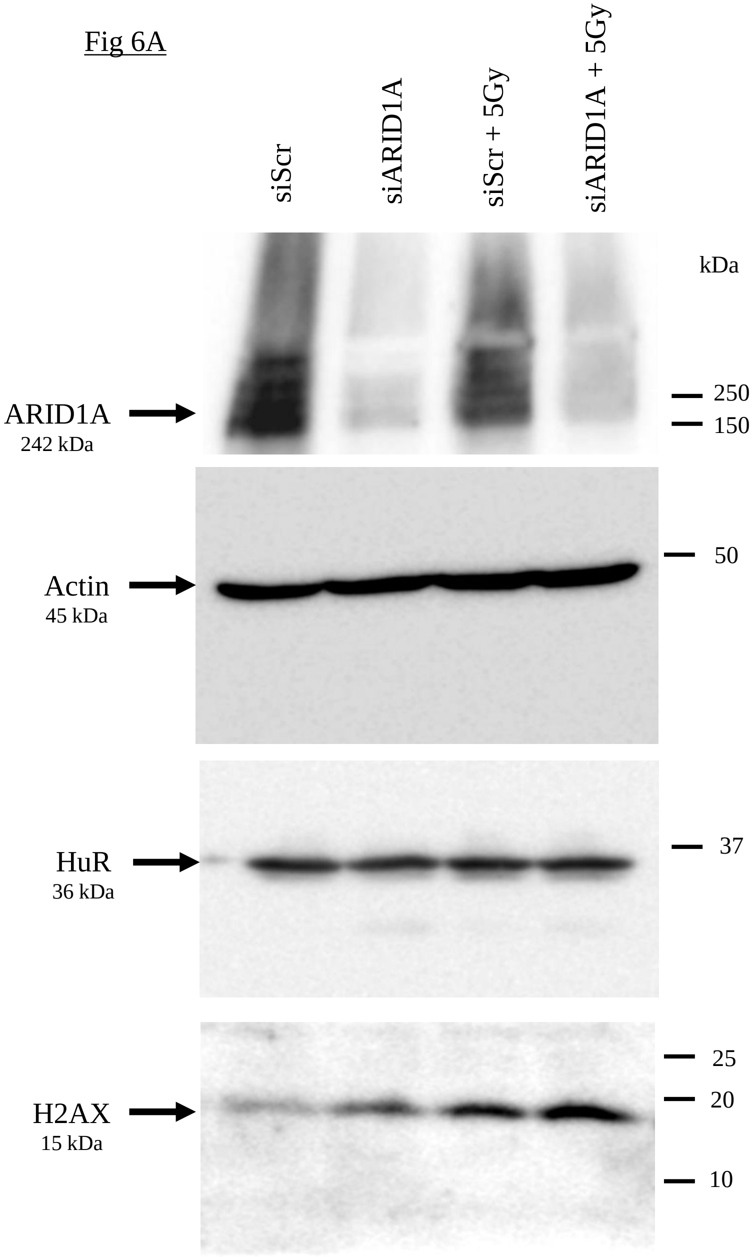

ARID1A	1	0.2	0.7	0.2
γH2AX	1	3.2	4.7	8
HuR	1	1	0.9	1
Actin	1	1	1	1

Fig 6C

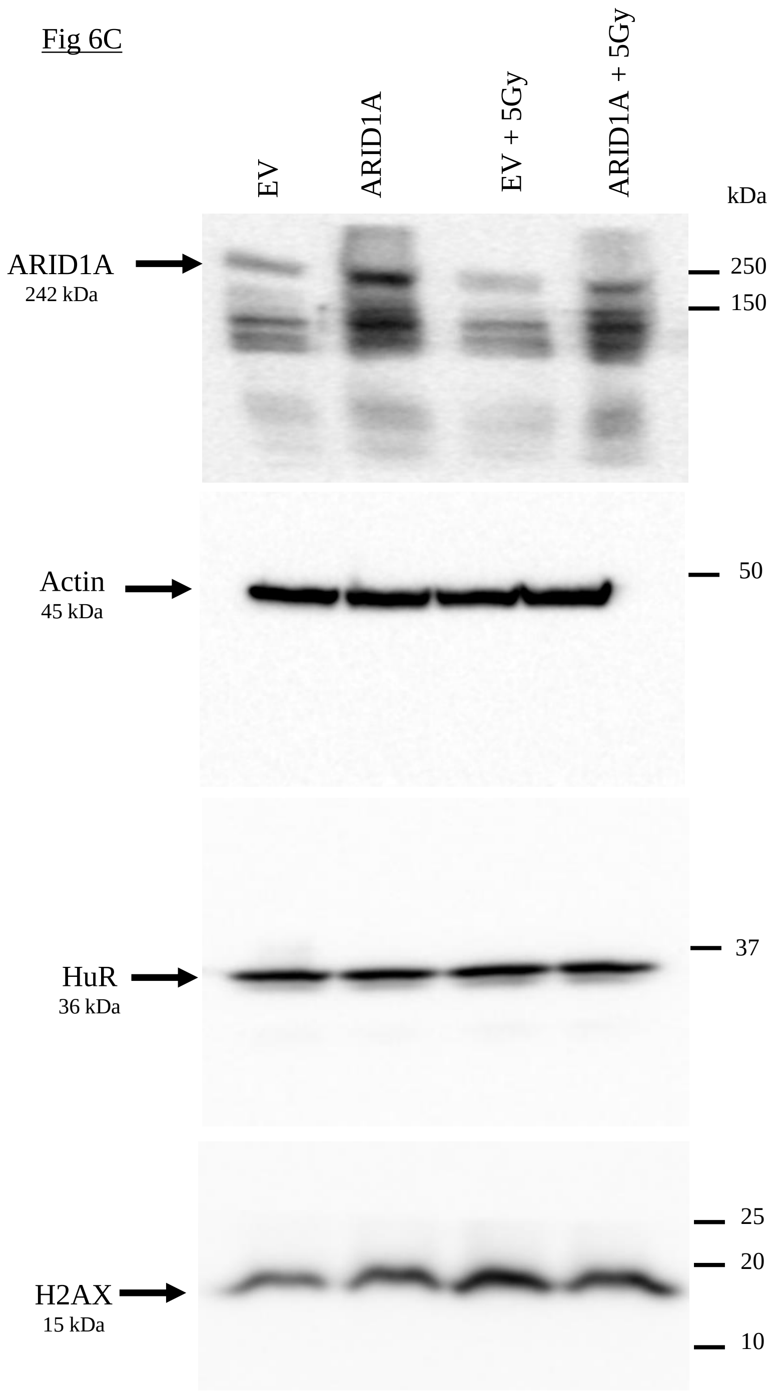

	EV	ARID1A	EV + 5Gy	ARID1A + 5Gy
ARID1A	1	2.1	1	1.5
γH2AX	1	1.7	2.8	1.6
HuR	1	1	1.1	0.9
Actin	1	1	1	1
